# Boosting Diffusion Networks with Deep External Context-Aware Encoders for Low-Light Image Enhancement

**DOI:** 10.3390/s25237232

**Published:** 2025-11-27

**Authors:** Pengliang Tang, Yu Wang, Aidong Men

**Affiliations:** School of Artificial Intelligence, Beijing University of Posts and Telecommunications, Beijing 100876, China; tangpengliang@bupt.edu.cn (P.T.); wywork@bupt.edu.cn (Y.W.)

**Keywords:** low-light image enhancement, diffusion models, context modeling, hybrid Transformer–Convolution blocks, CIELAB space

## Abstract

Low-light image enhancement (LLIE) requires modeling spatially extensive and interdependent degradations across large pixel regions, while directly equipping diffusion-based LLIE with heavy global modules inside the iterative denoising backbone leads to prohibitive computational overhead. To enhance long-range context modeling without inflating the per-step cost of diffusion, we propose ECA-Diff, a diffusion framework augmented with a deep External Context-Aware Encoder (ECAE). A latent-space context network built with hybrid Transformer–Convolution blocks extracts holistic cues from the input, generates multi-scale context features once, and injects them into the diffusion backbone as lightweight conditional guidance across all sampling steps. In addition, a CIELAB-space Luminance-Adaptive Chromaticity Loss regularizes conditional diffusion training and mitigates the cool color cast frequently observed in low-luminance regions. Experiments on paired and unpaired benchmarks show that ECA-Diff consistently outperforms recent state-of-the-art LLIE methods in both full-reference (PSNR/SSIM/LPIPS) and no-reference (NIQE/BRISQUE) metrics, with the external context path introducing only modest overhead relative to the baseline diffusion backbone. These results indicate that decoupling global context estimation from the iterative denoising process is an effective way to boost diffusion-based LLIE and provides a general compute-once conditioning paradigm for low-level image restoration.

## 1. Introduction

Images captured under low-light conditions often suffer from low signal-to-noise ratios (SNRs) and poor visibility because of the limited number of photons reaching the image sensor. These degradations compromise the usability of low-light images in both individual and industrial contexts, and constrain the performance of high-level vision-based downstream tasks. Although physics-based solutions can alleviate these issues to some extent, they suffer from inherent limitations: long exposure requires static scenes, high ISO induces severe noise, and high-end sensors are costly. As a result, algorithmic approaches to addressing the task of low-light image enhancement (LLIE) have become efficient and essential alternatives.

Due to the ill-posed nature of degraded low-light images, accurately restoring color and structural details remains a long-standing challenge. Over the years, a wide range of LLIE methods have been developed. Traditional approaches, such as histogram equalization (HE) [1,2] and Retinex-based methods [3,4], primarily focus on adjusting brightness, tone, or contrast to align with human visual perception while neglecting the presence of noise, which makes them ineffective under extremely low illumination or short exposure conditions. The recent comprehensive development of deep learning-based methods has significantly advanced the field of LLIE. Chen et al. [5] propose reconstructing high-quality images from extreme low-light raw-format sensor data with an end-to-end neural network to deal with the full low-light image processing pipeline. Following this work, a variety of works such as [6,7,8,9,10], apply end-to-end deep neural networks to handle LLIE tasks. At the same time, a series of methods combine the design of deep networks with the Retinex theory, e.g., [11,12,13]. Also, many state-of-the-art deep learning-based technologies have been introduced into this field, such as transformer [14,15,16,17], normalizing flow [18] and diffusion models [19,20,21,22,23]. Among these, diffusion models have recently emerged as a promising direction in LLIE due to their superior generative capabilities. The inherent denoising property of diffusion models makes them particularly effective in handling the noise prevalent in LLIE, yielding perceptually pleasing and smooth results that often outperform other learning-based methods.

However, most diffusion-based LLIE methods retain the DDPM [24] convention of a shallow U-Net [25] as the denoising backbone. Such backbones are efficient at estimating manually added white (i.i.d.) Gaussian noise but, due to their limited receptive field, struggle to capture the non-local and interdependent degradations characteristic of low-light scenes. Consistent with this, CNN-based studies [6,9,10] show that long-range, context-aware representations are essential for globally lifting illumination, suppressing widespread noise, recovering locally missing details, and maintaining color consistency. Recent diffusion variants illuminate the trade-off from two directions. PyDiff [19] demonstrates that conditional diffusion without explicit global correction can exhibit color bias; it mitigates this via an auxiliary refinement network executed alongside the denoiser at each sampling step, an approach that ties global correction to the iterative loop and thus scales inference cost with the number of steps. Conversely, CLE [21] injects a global target, namely image-level mean intensity, as exposure guidance, achieving accurate overall brightness, but the absence of explicit long-range modeling limits large-scale color consistency and fine-detail recovery. Taken together, these observations expose a central gap: diffusion-based LLIE needs global context that is both effective and decoupled from per-step inference, enabling large-scale structural and color correction without prohibitive computational overhead.

To this end, we propose *Boosting Diffusion Networks with a Deep External Context-Aware Encoder* (ECA-Diff) for LLIE, a framework that augments diffusion backbones with an External Context-Aware Encoder (ECAE) to strengthen global context modeling. During inference, ECAE is executed once per image and its features are reused at every sampling step, supplying stable global guidance with negligible per-step overhead. Inspired by Latent Diffusion Models (LDMs) [26], ECAE models global context in a compact latent space to extract information efficiently, and the resulting context-aware features are injected into the diffusion backbone as conditional guidance. Unlike LDMs, our denoising backbone operates in the full-resolution image space to preserve the fidelity required for LLIE. By leveraging the rich low-dimensional representations from ECAE, ECA-Diff broadens the contextual receptive field and extends the semantic capacity of the denoiser backbone, enabling more accurate modeling of long-range structures and more faithful restoration of global color distributions in LLIE tasks.

Specifically, the ECAE in ECA-Diff comprises a pretrained VAE [26] encoder that maps the input image to a compact latent space, followed by a Latent Context Network (LCN) to extract higher-level global context efficiently. Each LCN stage stacks multiple Hybrid Transformer–Convolution (HTC) blocks, in which a Transformer branch and a large-kernel convolutional branch run in parallel and are subsequently fused via Bidirectional Cross-Attention (Bi-CA). This hybrid design in latent space supplies global context-aware guidance while preserving fine-grained spatial cues, without incurring excessive computational cost. For multi-scale guidance, the LCN features are progressively upsampled, temporally modulated by the current diffusion timestep, and concatenated with the backbone features, thereby seamlessly injecting global context into the denoising trajectory.

In addition, we introduce an auxiliary color regularizer, the *Luminance-Adaptive Chromaticity Loss (LACL)*, to curb the cool, low-saturation cast frequently observed in low-luminance regions when training solely with the conditional noise objective and standard RGB-domain losses. LACL is computed in the CIELAB [27] space, leveraging its near-perceptual uniformity to decouple lightness from color, and imposes two simple constraints with a luminance-adaptive weighting: a hue-angle consistency term and a relative anti-desaturation term that penalizes chroma deficits without over-saturating bright areas. This auxiliary loss complements our main objective with negligible overhead and yields more faithful global color reproduction across scenes.

Extensive experiments on both paired and unpaired LLIE benchmarks show that ECA-Diff delivers consistent gains over state-of-the-art methods in quantitative metrics and perceived visual quality. The main contributions of this work are summarized as follows:

- We propose ECA-Diff, a diffusion-based LLIE framework that augments the diffusion backbone with an External Context-Aware Encoder to enhance long-range modeling. The encoder is executed once per image and reused across all sampling steps, thereby strengthening global color restoration and structural fidelity with negligible per-step overhead.
- We design an LCN that operates in a compact latent space to extract context-aware features for the diffusion backbone. The LCN comprises HTC blocks in which Transformer and large-kernel CNN branches run in parallel and are fused via bidirectional cross-attention, enabling strong global representation learning while preserving local detail with low computational overhead.
- We introduce LACL, an auxiliary loss in the CIELAB space that regularizes diffusion training by enforcing hue-angle consistency and penalizing desaturation via luminance-adaptive weighting. It improves global color fidelity and enhances chroma reconstruction in dark regions for LLIE.

## 2. Related Work

### 2.1. Traditional LLIE Methods

Typical traditional techniques focus on manipulating the global brightness and contrasts of images. The most classic families are HE-based and Retinex-based methods. HE-based LLIE methods use the cumulative distribution function to adjust the output gray levels to enhance hidden details in dark areas. The early HE-based methods conduct statistical operations based on the gray values of the whole image [1,28], leading to poor local enhancement performance, as well as over-exposure or under-exposure issues. To solve these problems, HE methods based on local regions and other different prior constraints are proposed [2,29,30,31]. Retinex-based LLIE methods are based on the Retinex theory of color vision [3], the essence of which is to determine the reflection map of an image by removing the effects of the low-light illumination map from the image. Then obtain the enhanced image by integrating the enhanced illumination map and the retained reflection map. Lee et al. [32] first introduces the Retinex theory into image enhancement tasks. Following this, a series of subsequent studies design more refined structures and hand-crafted priors to achieve satisfactory LLIE results, e.g., multi-scale Retinex [33], robust Retinex [34], structure aware prior [4], joint intrinsic–extrinsic prior [35].

HE and classical Retinex methods are constrained by hand-crafted intensity-domain priors and locality, so under extreme low illumination they often fail to recover missing detail or faithful chroma. Their core ideas, namely Retinex’s separation of illumination and reflectance and HE’s global tone and contrast normalization, have nevertheless informed many learning-based LLIE methods. In this paper, ECA-Diff adopts this global information perspective while overcoming the brittleness of classical pipelines.

### 2.2. Deep Learning-Based LLIE Methods

Since LLNet [36], many deep learning approaches for LLIE have been developed [5,6,7,8,9,10,11,12,13]. These methods rely on carefully designed architectures, tailored training strategies, and, most importantly, paired supervision that learns a mapping from low-light to normal-light images. Several works among them combine deep networks with Retinex-inspired decomposition to improve illumination adjustment and degradation removal [11,12,13]. Advanced backbones and priors have also been introduced, including transformer-based models [14,15,16,17,37], the normalizing-flow method LLFlow [18], and diffusion-based approaches [19,20,21,22,23], which we detail in the next subsection.

In the circumstances of global information construction, GIANet [9] augments convolution with global-mean features to mitigate color inconsistency, and Zhang et al. [6] combine non-local operations with channel attention to reduce color artifacts. Other advanced models such as MIRNet [38] and IAT [37] explore hierarchical or transformer-based modules to capture long-range dependencies and global context. Despite the progress, simple global cues lack broad adaptability, while more sophisticated global computation often increases complexity. In contrast, our LCN operates in a compact latent space and uses HTC blocks to extract context-aware representations that balance global semantics and local details. In HTC blocks, the transformer branch and the large-kernel CNN branch run in parallel and are fused via bidirectional cross-attention, which improves efficiency. Unlike CoAtNet [39], which integrates convolution and transformer attention throughout the full network, the HTC block is a lightweight plug-in that works at lower-resolution latent scales, enabling deeper context encoding without significant overhead. Compared with ConvFormer [40], which fuses convolution and transformer layers sequentially within each block, HTC adopts a reciprocal parallel-fusion strategy that increases flexibility and feature diversity.

### 2.3. Diffusion Models for LLIE

In recent years, diffusion-based generative models, in particular denoising diffusion probabilistic models (DDPMs) [24], have demonstrated strong potential across image restoration tasks, including super-resolution [26,41], inpainting [42,43], and shadow removal [44]. Several recent studies have adapted diffusion to LLIE [19,20,21,22,23]. For instance, CLE [21] enables user-specified brightness and region-aware enhancement by injecting a unified illumination embedding into the denoising backbone and leveraging Segment Anything Model [45] guided spatial prompts. PyDiff [19] accelerates inference with a pyramid sampling scheme that progressively increases resolution within a single reverse process and introduces a global corrector to mitigate color and other global degradations, yielding improved efficiency and robustness. AnlightenDiff [22] employs a dynamically regulated anchoring mechanism and sampler to constrain iterative refinement to remain faithful to the input. DiffLL [20] adopts wavelet-domain conditional diffusion to speed up inference while preserving visual quality via dedicated high-frequency modules. SeparatedDiff [23] follows a Retinex-inspired decomposition and trains separate diffusion models for illumination enhancement and detail generation.

Compared with prior diffusion-based LLIE methods, ECA-Diff focuses on strengthening the diffusion backbone’s global context modeling to improve illumination enhancement, structural recovery, and color consistency. Unlike SeparatedDiff [23], which trains separate diffusion branches, and PyDiff [19], which couples context modeling with the iterative denoising steps and increases per-step computation, ECA-Diff introduces a plug-in External Context-Aware Encoder that supplies rich, image-conditioned global features to the diffusion backbone without entangling them with the iterative process. The encoder runs once per image; its low-dimensional features are temporally modulated and injected across scales, and the denoiser operates in full-resolution image space. This decoupled design enables efficient global illumination restoration and structure recovery while minimizing the additional per-step cost typical of tightly integrated or multi-stage architectures.

## 3. Methodology

This section details ECA-Diff, an External Context-Aware Encoder (ECAE) guided diffusion framework for low-light image enhancement (LLIE). We begin by reviewing the diffusion formulation, then outline the overall ECA-Diff pipeline to provide a high-level workflow. Next, we describe each component in turn, including the ECAE, the Latent Context Network (LCN), the Hybrid Transformer–Convolution (HTC) block augmented with Bidirectional Cross-Attention (Bi-CA), and the Time-modulated Context Fusion (TCF) block. Subsequently, we introduce the Luminance-Adaptive Chromaticity Loss (LACL) and specify the overall training objective and inference strategy. Finally, we present the datasets and the evaluation protocol used in our experiments.

### 3.1. Preliminaries: Diffusion Models

**Denoising Diffusion Probabilistic Model (DDPM).** Diffusion models are a class of generative models that synthesize data by learning to invert a gradual Gaussian noising process, progressively transforming noise into samples from the target distribution. The classic DDPM [24] instantiates this idea with a fixed, typically increasing variance schedule ${\left\{\beta_{t}\right\}}_{t=1}^{T}\subset \left(0,1\right)$ and a noise-prediction network $\epsilon_{\theta }$ that parameterizes the reverse process. The forward process gradually adds Gaussian noise to a clean image $y_{0}$:

(1) $$q\left(y_{t}|y_{t-1}\right)=N\;\left(y_{t};\sqrt{\alpha_{t}}\;y_{t-1},\;\left(1-\alpha_{t}\right)I\right),$$

where $\alpha_{t}=1-\beta_{t}$ and I is the identity matrix. Let ${\bar{\alpha }}_{t}=\prod_{i=1}^{t}\alpha_{i}$, with ${\bar{\alpha }}_{0}=1$; then the marginal distribution $q(y_{t}|y_{0})$ can be expressed as

(2) $$q\left(y_{t}|y_{0}\right)=N\;\left(y_{t};\sqrt{{\bar{\alpha }}_{t}}\;y_{0},\;\left(1-{\bar{\alpha }}_{t}\right)I\right).$$

The variance schedule ${\left\{\beta_{t}\right\}}_{t=1}^{T}$ is carefully designed such that ${\bar{\alpha }}_{t}\to 0$ as t→T, and hence $q\left(y_{T}\right)\approx N\left(0,I\right)$.

In the reverse process, a neural network $\epsilon_{\theta }$ is trained to predict the noise component to progressively convert the standard Gaussian into the target data distribution:

(3) $$p_{\theta }\left(y_{t-1}|y_{t}\right)=N\;\left(y_{t-1};\mu_{\theta }\left(y_{t},t\right),\;{\tilde{\beta }}_{t}I\right),$$

where

(4) $$\mu_{\theta }\left(y_{t},t\right)=\frac{1}{\sqrt{\alpha_{t}}}\;\left(y_{t}-\frac{\beta_{t}}{\sqrt{1-{\bar{\alpha }}_{t}}}\;\epsilon_{\theta }\left(y_{t},t\right)\right),\;{\tilde{\beta }}_{t}=\frac{1-{\bar{\alpha }}_{t-1}}{1-{\bar{\alpha }}_{t}}\;\beta_{t}.$$

During inference, the reverse diffusion process starts by sampling $y_{T}∼N\left(0,I\right)$ and then iteratively applies the learned denoising transitions to recover a clean image $y_{0}$.

**Training objective.** Given standard Gaussian noise ϵ∼N(0,I) and the noisy sample at time *t* generated by the forward diffusion, $y_{t}=\sqrt{{\bar{\alpha }}_{t}}\;y_{0}+\sqrt{1-{\bar{\alpha }}_{t}}\;\epsilon$, a noise-prediction network $\epsilon_{\theta }\left(y_{t},t\right)$ is trained to estimate ϵ. The parameters θ are optimized by minimizing a simplified noise-prediction loss:

(5) $$L_{\mathrm{ddpm}}=E_{y_{0},\;t,\;\epsilon }\;\left[{∥\;\epsilon -\epsilon_{\theta }\;\left(y_{t},\;t\right)∥}_{2}^{2}\right].$$

**Denoising Diffusion Implicit Model (DDIM) Sampling.** With the non-stochastic (η=0) setting, where η∈[0,1] controls the stochasticity of the generalized reverse update, DDIM [46] renders the reverse diffusion deterministic by conditioning the update on a fixed signal trajectory, thereby removing stochastic noise injection and accelerating sampling. The update rule is given by

(6) $$y_{t-1}=\sqrt{{\bar{\alpha }}_{t-1}}\left(\frac{y_{t}-\sqrt{1-{\bar{\alpha }}_{t}}\;\epsilon_{\theta }\left(y_{t},t\right)}{\sqrt{{\bar{\alpha }}_{t}}}\right)+\sqrt{1-{\bar{\alpha }}_{t-1}}\;\epsilon_{\theta }\left(y_{t},t\right).$$

In this work, we adopt DDIM for inference, while training strictly follows the standard DDPM strategy with the simplified noise-prediction objective in Equation (5).

### 3.2. External Context-Aware Encoder Guided Diffusion Model

High-fidelity low-light image reconstruction requires preserving fine details throughout the generative process. While latent diffusion models (LDMs) [26] achieve efficiency by performing denoising in a compressed latent space, their autoencoder compression and latent-space processing can discard fine structural details. Therefore, in this work, we employ a diffusion backbone that operates directly in pixel space at the native resolution, thereby maintaining richer visual information for subsequent reconstruction. In addition to preserving fine details, effective LLIE also demands robust modeling of global context to ensure color consistency, global denoising, and coherent illumination restoration. However, achieving this within diffusion models via internal global-context modules typically requires complex components, which substantially increase the per-step computational cost, which accumulates over sampling steps to raise the total inference time. To address these challenges, we propose *ECA-Diff*, a standard denoising diffusion backbone enhanced with an *External Context-Aware Encoder* (ECAE). This encoder is run once per image to compute deep multi-scale latent condition features that are subsequently reused across all sampling steps, thereby providing robust global context guidance with minimal per-step overhead.

**Figure 1:**
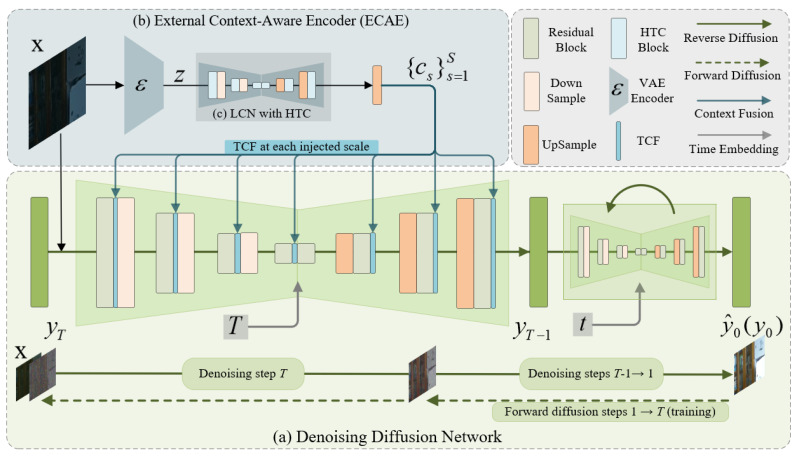
Architecture of ECA-Diff. LCN denotes the Latent Context Network; TCF denotes time-modulated context fusion block. The timestep *t* is embedded at each backbone stage and jointly used in the TCF modules to modulate the fused features. (**a**) Denoising Diffusion Network: starting from $y_{T}\;∼\;N\left(0,I\right)$, each step takes $\left(y_{t},x,t\right)$ and the context features $c_{s}$ from ECAE, predicts noise $\epsilon_{\theta }\left(y_{t},t\right)$, and updates the state to $y_{t-1}$. (**b**) External Context-aware Encoder (ECAE): the input x is encoded once by a pretrained VAE E to produce a latent *z*; (**c**) within (**b**), the LCN (built with HTC blocks) converts *z* into multi-scale features ${\left\{\;c_{s}\;\right\}}_{s=1}^{S}$, which are cached and reused at every sampling step by the TCFs.

As illustrated in Figure 1, our system comprises a denoising diffusion backbone and an external encoder ECAE. The backbone follows the standard DDPM formulation [24]; following prior practice [21], we implement a conditional variant in which the U-Net [25] denoiser receives the low-light input x together with the time embedding *t* and predicts the noise $\epsilon_{\theta }\left(y_{t},t,x\right)$. For inference, we adopt DDIM [46] (non-stochastic, η = 0) to deterministically update from $y_{T}$ to $y_{0}$. In addition to conditioning on x, ECAE constructs a time-invariant set of multi-scale deep context features ${\left\{c_{s}\right\}}_{s=1}^{S}$, where *S* matches the number of backbone resolution scales. These features are computed once per image and cached before sampling begins, and then injected at matching resolutions via TCF blocks. The TCF block comprises a time-modulation function $g_{s}\left(c_{s},t\right)$ that synchronizes the injected features with the reverse diffusion process, yielding the conditional denoiser $\epsilon_{\theta }\left(y_{t},t,x,C\right)$, where $C\;≜\;{\left\{\;g_{s}\left(c_{s},t\right)\;\right\}}_{s=1}^{S}.$

Given the low-light input x, the target normal-light image $y_{0}$, and the cached time-modulated context C, the training objective for noise prediction is

(7) $$L_{\mathrm{ddpm}}^{\mathrm{cond}}=E_{y_{0},\;t,\;\epsilon }\;\left[{∥\;\epsilon -\epsilon_{\theta }\;\left(y_{t},t,x,C\right)∥}_{2}^{2}\right],\;y_{t}=\sqrt{{\bar{\alpha }}_{t}}\;y_{0}+\sqrt{1-{\bar{\alpha }}_{t}}\;\epsilon ,\;\epsilon \;∼\;N\left(0,I\right).$$

The DDIM-style conditional reverse update is

(8) $$y_{t-1}=\sqrt{{\bar{\alpha }}_{t-1}}\left(\frac{y_{t}-\sqrt{1-{\bar{\alpha }}_{t}}\;\epsilon_{\theta }\left(y_{t},t,x,C\right)}{\sqrt{{\bar{\alpha }}_{t}}}\right)+\sqrt{1-{\bar{\alpha }}_{t-1}}\;\epsilon_{\theta }\left(y_{t},t,x,C\right).$$

### 3.3. External Context-Aware Encoder

The ECAE is designed to externally enhance the backbone diffusion model by supplying deep context-aware representations, thereby mitigating color shifts and improving overall LLIE performance. As shown in Figure 1b, the ECAE consists of three components: (i) a pretrained latent encoder that maps the input x into a compact latent code *z*; (ii) a lightweight LCN composed of HTC blocks that extract both long-range and local cues from *z* and transform them into multi-scale context-aware features ${\left\{c_{s}\right\}}_{s=1}^{S}$; and (iii) a time-modulated fusion module, TCF, that injects these features into the backbone denoiser.

#### 3.3.1. Pre-Trained Latent Encoder

Although a latent-space diffusion scheme is not adopted in the ECA-Diff backbone to avoid potential information loss, the ECAE is specifically designed for global context modeling. In this setting, employing the pretrained VAE encoder from LDM [26] provides a lightweight and computationally efficient solution for information integration and dimensionality reduction. Benefiting from large-scale pretraining (e.g., on ImageNet [47]), this encoder produces semantically rich latent features without incurring substantial additional training cost. Architecturally, the VAE encoder in LDM comprises a convolutional downsampling path augmented with residual blocks and a self-attention layer at the bottleneck. This design helps preserve essential local structures while capturing a degree of global dependencies, albeit with some inevitable loss of fine-grained details. Given a low-light image x, the encoder outputs a latent feature map formulated as

(9) z=E(x),

which matches the spatial resolution of the backbone denoiser’s bottleneck and has four channels. The latent code z thus provides a compact representation that facilitates deeper context modeling in the subsequent LCN module.

Note that the pretrained VAE encoder is frozen during the early stages of ECA-Diff training to fully exploit the priors learned from large-scale pretraining; we subsequently unfreeze it and fine-tune with a smaller learning rate to better adapt to the target dataset and the backbone network.

#### 3.3.2. Latent Context Network

The latent representation z produced by the preceding VAE encoder provides an initial feature map at low spatial resolution (one-eighth of the input size) with four channels, and remains compatible with the ECA-Diff backbone bottleneck. To construct deeper and more comprehensive global context, we introduce the *Latent Context Network* (LCN), which employs a U-Net [25] with two levels of downsampling and upsampling, augmented with skip connections to preserve spatial details, as illustrated in Figure 1c. Each stage of the LCN is built upon multiple HTC blocks, which serve as the primary computational units for extracting rich global context features (detailed in the next subsection).

To effectively leverage the deep context features extracted by the LCN for guiding the backbone in predicting the noise $\epsilon_{\theta }$ for a given low-light input x, we generate and inject multi-scale context features into the corresponding stages of the denoiser. Specifically, the LCN first produces a stage-1 context feature map $c_{1}$, formulated as

(10) $$c_{1}=\mathrm{LCN}\left(z\right).$$

Subsequently, a series of transposed convolutions with a 2× upsampling factor are applied to generate multi-scale context features ${\left\{c_{s}\right\}}_{s=1}^{S}$ that are spatially aligned with the backbone stages.

#### 3.3.3. Hybrid Transformer–Convolution Block

The proposed *Hybrid Transformer–Convolution* (HTC) block serves as the core computational unit of each LCN stage, designed to integrate the complementary strengths of convolutional and transformer-based representations. Operating in the low-resolution, low-channel latent space generated by the VAE encoder, HTC blocks efficiently capture both long-range dependencies and fine-grained local patterns by combining large-kernel convolutions with pixel-level self-attention.

**Figure 2:**
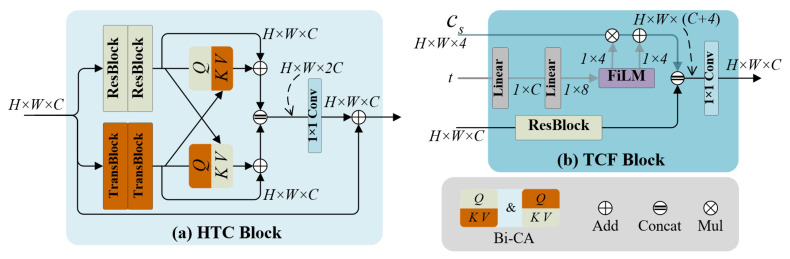
HTC and TCF blocks. (**a**) HTC: A Hybrid Transformer–Convolution block with bidirectional cross-attention (Bi-CA). Q/KV box colors match their source branch. Queries Q from one branch attend to key–value pairs (K,V) from the other branch; the two branches exchange attention, and their outputs are fused via concatenation followed by a 1×1 convolution. (**b**) TCF: time-modulated context fusion. The stage-*s* context map $c_{s}\;\in \;R^{H\times W\times 4}$ is modulated by FiLM-style, time-conditioned scale and shift, then concatenated with the backbone features and compressed to H×W×C. Symbols denote elementwise addition (Add), concatenation (Concat), and multiplication (Mul).

As illustrated in Figure 2a, the HTC adopts a parallel architecture consisting of a convolution branch and a transformer branch. The convolution branch comprises two residual blocks with relatively large kernels, thereby expanding the receptive field while preserving local detail fidelity. The transformer branch includes two self-attention blocks that specialize in modeling global dependencies across spatial positions. To promote mutual feature refinement between the two branches, *Bidirectional Cross-Attention* (Bi-CA) is applied: convolution-branch queries are refined using transformer-derived keys and values, while transformer-branch queries are refined using CNN-derived keys and values. This reciprocal interaction allows each branch to benefit from the complementary information of the other, leading to richer and more context-aware representations. Compared with naive element-wise addition or channel concatenation, which mix features in a fixed manner, Bi-CA performs content-adaptive, spatially varying fusion so that each location selectively aggregates the most informative responses from the complementary branch. After the cross-attention step, the enhanced convolutional and transformer features are concatenated along the channel dimension, fused by a 1×1 convolution, and added to the original input through a residual connection. This fusion strategy consolidates multi-scale contextual cues while stabilizing training by preserving the original signal pathway. In subsequent stages of the LCN, stacked HTC blocks progressively enrich the latent representation, providing robust and semantically meaningful context features to guide the ECA-Diff backbone.

#### 3.3.4. Time-Modulated Context Fusion into the Denoiser

Following a conditioning paradigm similar to LDM [26], we inject multi-scale image features into the backbone denoiser. The proposed *Time-modulated Context Fusion (TCF)* block is illustrated in Figure 2b. To balance efficiency and accuracy, TCF replaces cross-attention with lightweight, concatenation-based fusion. To synchronize guidance with the reverse diffusion process, we apply feature-wise linear modulation (FiLM) [48]: given the backbone’s time embedding *t*, small modulators predict per-channel scale and shift, producing step-aware context ${\tilde{c}}_{s}=g_{s}\left(c_{s},t\right)$. At each stage *s*, ${\tilde{c}}_{s}$ is concatenated with the backbone activations and compressed with a 1×1 projection to match dimensionality before being passed through the denoiser. This design provides global illumination and color guidance while preserving local detail, with only a small additional per-step cost. Denoting the condition set as (x,C), where $C={\left\{{\tilde{c}}_{s}\right\}}_{s=1}^{S}$, the conditional reverse diffusion follows Equations (3) and (4) with the noise predictor $\epsilon_{\theta }\left(y_{t},t,x,C\right)$.

### 3.4. Luminance-Adaptive Chromaticity Loss

Optimizing only the conditional noise-prediction objective $L_{\mathrm{ddpm}}^{\mathrm{cond}}$ in Equation (7) on paired low-light data is, in principle, sufficient to train ECA-Diff for high-quality LLIE. However, in practice we consistently observe a *cool color cast with reduced saturation*, most notably in low-luminance regions. This artifact often persists even when auxiliary RGB-domain losses are applied to the denoised estimate ${\hat{y}}_{0}$, including pixelwise $\ell_{1}$, structural similarity index measure (SSIM) [49], and a perceptual term [50]. To explicitly regularize color tone, we introduce the *Luminance-Adaptive Chromaticity Loss (LACL)*, computed in the CIELAB (L*a*b*) space [27]. Owing to CIELAB’s near-perceptual uniformity, lightness is decoupled from chromatic components, enabling precise constraints on hue and chroma. LACL complements RGB-domain losses and, in this work, consists of two terms: (i) a hue-angle consistency term and (ii) a relative anti-desaturation term that penalizes chroma deficits. In addition, we apply a luminance-adaptive weight that increases as the target lightness decreases, thereby reinforcing color reconstruction in darker regions while avoiding unnecessary penalties in bright areas. We thus define LACL as follows.

Let ${\hat{y}}_{0},y\;\in \;{\left[0,1\right]}^{3\times H\times W}$ denote the predicted and reference *sRGB* images. We convert them to CIELAB: $\left(L_{p}^{*},a_{p}^{*},b_{p}^{*}\right)=\mathrm{Lab}\left({\hat{y}}_{0}\right);\left(L_{g}^{*},a_{g}^{*},b_{g}^{*}\right)=\mathrm{Lab}\left(y\right).$ Here, $L^{*}$ is perceptual lightness (0 for black, 100 for white), $a^{*}$ encodes the green (−) ↔ red (+) axis, and $b^{*}$ encodes the blue (−)↔ yellow (+) axis; subscripts *p* and *g* refer to predicted and ground-truth quantities, respectively. We define chroma $C^{*}=\sqrt{a^{*2}+b^{*2}}$. Throughout, 〈·〉 denotes the per-image spatial average, i.e., $\langle f\rangle \;=\;\frac{1}{HW}\sum_{i=1}^{H}\sum_{j=1}^{W}f_{ij}$.

**Hue loss via unit-vector cosine.** We measure a cosine mismatch between unit-normalized chromaticity vectors to penalize hue discrepancies:

(11) $$L_{\mathrm{hue}}=\langle \;w_{\mathrm{hue}}\left(1-\cos\;\left(\frac{\left(a_{p}^{*},\;b_{p}^{*}\right)}{C_{p}^{*}+\varepsilon },\;\frac{\left(a_{g}^{*},\;b_{g}^{*}\right)}{C_{g}^{*}+\varepsilon }\right)\right)\rangle .$$

Here cos(·,·) is the cosine similarity between two 2D vectors, and ε>0 prevents division by zero and stabilizes near-gray regions ($C^{*}\;\approx 0$).

**Desaturation penalty.** To discourage chroma collapse while avoiding over-saturation, we use

(12) $$L_{\mathrm{desat}}=\langle \;w_{\mathrm{desat}}\;\frac{\max\;\left(0,\;C_{g}^{*}-C_{p}^{*}\right)}{C_{g}^{*}+\varepsilon }\rangle .$$

**Luminance- and chroma-aware weights.** We emphasize darker and more colorful regions while de-emphasizing near-gray pixels using the scalar gates defined as follows:

(13) $$w_{\mathrm{hue}}:=w_{L}\left(1-s_{C}\right),\;w_{\mathrm{desat}}:=w_{L}\;m\left(C_{g}^{*}\right).$$

In Equation (13), $w_{L}=0.5+0.5\;{\left(1-\frac{L_{g}^{*}}{100}\right)}^{\alpha_{L}}$, increasing the weight as luminance decreases; $s_{C}=\exp\;\left(-\frac{C_{g}^{*2}}{2\sigma_{C}^{2}}\right)$, close to 1 for near-gray regions ($C_{g}^{*}\;\approx \;0$) and decaying with chroma; $m\left(C_{g}^{*}\right)={\left(\frac{C_{g}^{*}}{C_{g}^{*}+\tau_{C}}\right)}^{\gamma_{C}}$, a monotonic saturating function of chroma where $\tau_{C}$ sets the knee and $\gamma_{C}$ the slope. Unless stated otherwise, hyperparameters are fixed across all experiments: $\alpha_{L}$ = 1.5, $\sigma_{C}$ = 20, $\tau_{C}$ = 7.5, $\gamma_{C}$ = 1.4, and ε = $10^{-6}$.

In summary, $w_{\mathrm{hue}}=w_{L}\left(1-s_{C}\right)$ focuses hue supervision on dark, chromatic pixels while downweighting near-gray regions, whereas $w_{\mathrm{desat}}=w_{L}\;m\left(C_{g}^{*}\right)$ strengthens anti-desaturation constraints in dark, highly saturated areas.

**Overall objective.** The LACL objective is finally defined as

(14) $$L_{\mathrm{LACL}}=\lambda_{h}\;L_{\mathrm{hue}}+\lambda_{d}\;L_{\mathrm{desat}},$$

where $\lambda_{h}$ = 1.0 and $\lambda_{d}$ = 0.8 by default.

### 3.5. Overall Optimization Objective

To stabilize training and improve convergence, we optimize the conditional objective $L_{\mathrm{ddpm}}^{\mathrm{cond}}$ together with *LACL* and three RGB-domain losses applied to ${\hat{y}}_{0}$. The auxiliary RGB losses are summarized below:

(i) Pixelwise loss: $L_{\mathrm{RGB}-\ell_{1}}=\langle ∥{\hat{y}}_{0}{-y∥}_{1}\rangle$;
(ii) Structural loss: $L_{\mathrm{SSIM}}=1-\mathrm{SSIM}\left({\hat{y}}_{0},y\right)$ [49];
(iii) Perceptual loss: $L_{\mathrm{perc}}=\sum_{l\in F}\langle ∥\phi_{l}\left({\hat{y}}_{0}\right)-\phi_{l}\left(y\right){∥}_{2}\rangle$ [50], where $\phi_{l}\left(\;\cdot \;\right)$ are VGG-16 features [51] pretrained on ImageNet [47], and F indexes a small set of layers.

Therefore, the final training objective is

(15) $$L_{\mathrm{train}}=L_{\mathrm{ddpm}}^{\mathrm{cond}}+\lambda_{\mathrm{RGB}-\ell_{1}}\;L_{\mathrm{RGB}-\ell_{1}}+\lambda_{\mathrm{SSIM}}\;L_{\mathrm{SSIM}}+\lambda_{\mathrm{perc}}\;L_{\mathrm{perc}}+\lambda_{\mathrm{LACL}}\;L_{\mathrm{LACL}}.$$

Here, $\lambda_{\mathrm{RGB}-\ell_{1}}$, $\lambda_{\mathrm{SSIM}}$, $\lambda_{\mathrm{perc}}$, and $\lambda_{\mathrm{LACL}}$ are scalar weights tuned on a validation set.

### 3.6. Datasets and Evaluation Protocol

**Datasets.** We adopt widely used LLIE benchmarks covering both paired and unpaired settings. The paired benchmarks include LOL [11] and LOL-v2 [52]. The LOL dataset comprises 500 real-captured low/normal-light pairs, of which 485 are used for training and 15 for testing. To assess performance on a larger and more diverse real-captured corpus, we additionally use the Real subset of LOL-v2, which contains 689 pairs for training and 100 pairs for testing. We follow the official splits for both datasets. To evaluate cross-dataset generalization, we also report results on several unpaired collections commonly used in LLIE, including DICM [2], LIME [4], MEF [53], NPE [54], and VV [55].

**Preprocessing and Augmentation.** For each paired sample, we apply identical augmentations—random 128 × 128 cropping and horizontal/vertical flips (*p* = 0.5). The low-light input and the reference image are both normalized to [0,1] and linearly remapped to [−1,1] to match the diffusion model’s working dynamic range. During evaluation, we disable cropping and other geometric transforms; numerical preprocessing remains identical to the training pipeline.

**Evaluation Metrics.** We evaluate with full-reference and no-reference image quality assessment (IQA) metrics for paired and unpaired datasets, respectively. Following common LLIE practice, we report Peak Signal-to-Noise Ratio (PSNR) and SSIM [49] on LOL and LOL-v2 Real datasets. PSNR measures fidelity by mapping the mean squared error (MSE) between the enhanced result and the reference onto a logarithmic decibel scale, while SSIM is a perception-oriented metric that jointly considers luminance, contrast, and structural consistency. Unless otherwise noted, all values are computed on 8-bit sRGB images, per image and then averaged over the test set. To better reflect human visual preference, we also report the Learned Perceptual Image Patch Similarity (LPIPS) [50] on paired datasets, using the official implementation with the AlexNet [56] backbone. This complements PSNR/SSIM by comparing deep feature representations across multiple scales. For unpaired datasets (DICM, LIME, MEF, NPE, VV), we evaluate using BRISQUE [57] and NIQE [58], two widely used no-reference IQA measures that correlate with human judgments without requiring ground-truth images. Briefly, BRISQUE (opinion-aware) regresses natural scene statistics features against human opinion scores, making it sensitive to artifacts such as noise, blur, and contrast shifts; NIQE (opinion-unaware) measures the deviation of an image’s statistics from a pristine natural-image model. We use standard implementations and report per-image scores averaged over each dataset.

**Implementation Details.** We implement all models in PyTorch 1.13.1 and train on a single NVIDIA RTX 4090 GPU. Unless noted, training uses mini-batches of 12 randomly cropped 256 × 256 patches. We adopt AdamW with weight decay $1\times 10^{-4}$ and three parameter groups: (i) the diffusion backbone with learning rate $lr=5\times 10^{-5}$, (ii) the LCN head with learning rate 10×lr, and (iii) the fine-tuned parameters of the pretrained VAE encoder with learning rate 0.5×lr. We apply cosine annealing over the full schedule with warm-up (multiplier 2.0) for the first 10% of the 8000 training epochs. A dropout rate of 0.15 is used in the network blocks, and the global gradient norm is clipped to 1.0 at each step.

For the diffusion setup, we use a 1000-step diffusion horizon with a linear β schedule from $1\times 10^{-4}$ to $2\times 10^{-2}$ during training. Unless otherwise stated, inference adopts DDIM sampling with 100 steps.

The denoising backbone takes a 6-channel input formed by concatenating the low-light RGB image and the current noisy latent $y_{t}$. The backbone is a U-Net [25] with a base channel width of 128 and channel multipliers [1,2,3,4]. It performs three downsampling and three symmetric upsampling operations, yielding four resolution scales and nine stages in total (encoder: 4; bottleneck: 1; decoder: 4), with two residual blocks per stage. Consequently, in the multi-scale conditioning set ${\left\{c_{s}\right\}}_{s=1}^{S}$ we use *S* = 4. The LCN within ECAE consumes the 4-channel latent produced by the pretrained VAE encoder E; it uses a base channel width of 64 with channel multipliers [2,4,4], and two HTC blocks are adopted per stage. During training, E is frozen for the first 2000 epochs and then fine-tuned with half the backbone learning rate. Loss terms and their default hyperparameters follow the corresponding subsection in the method section.

## 4. Results

**Figure 3:**
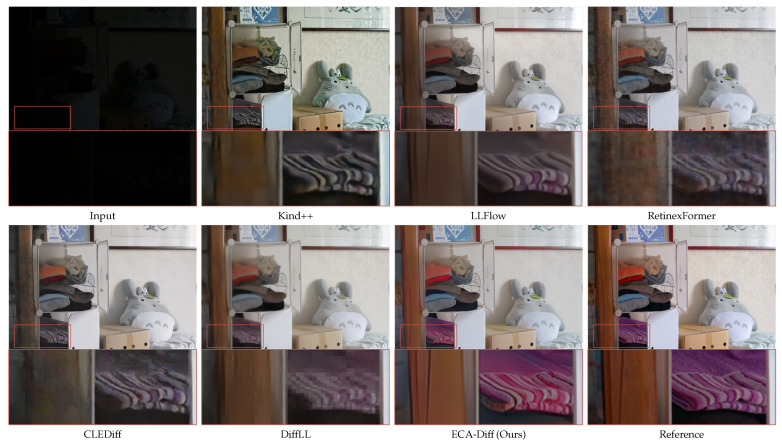

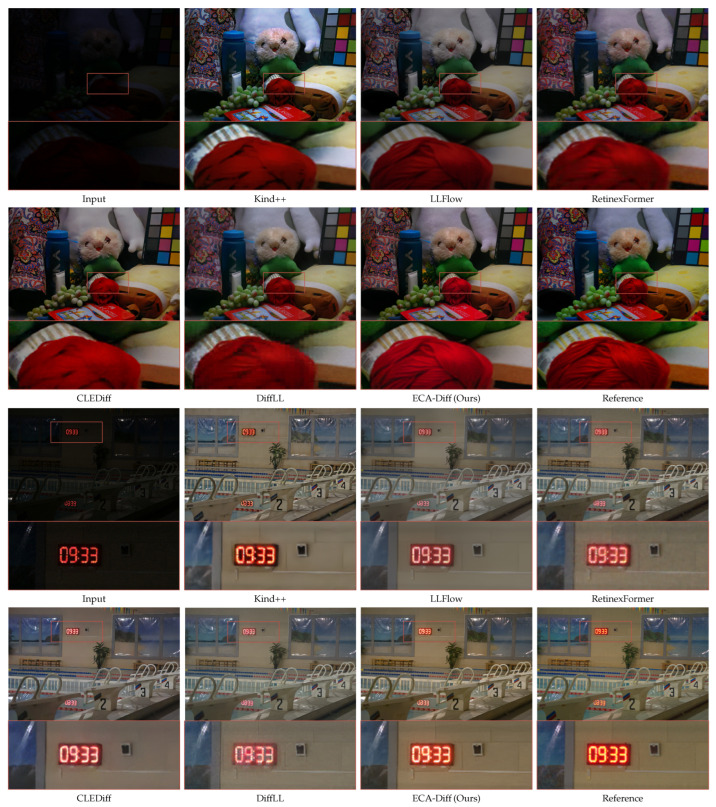
Visual comparison across multiple scenes on LOL dataset. Each group shows Input, Kind++ [13], LLFlow [18], RetinexFormer [16], CLEDiff [21], DiffLL [20], ECA-Diff (Ours), and the Reference. The region highlighted by the red box is enlarged below to show finer details. Please zoom in for best viewing.

**Figure 4:**
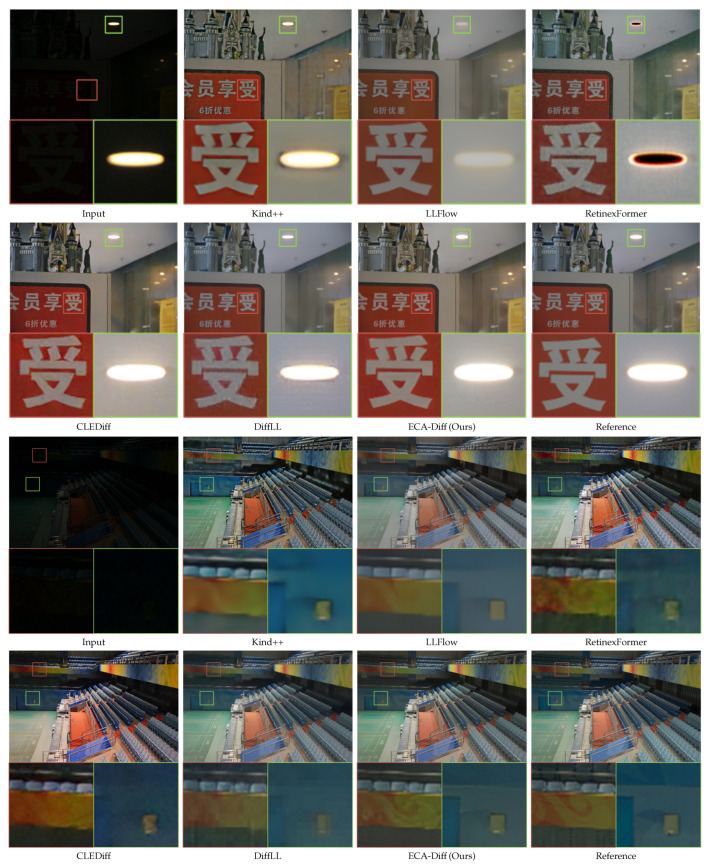
Visual comparison across multiple scenes on LOL-v2 Real dataset. Each group shows Input, Kind++ [13], LLFlow [18], RetinexFormer [16], CLEDiff [21], DiffLL [20], ECA-Diff (Ours), and the Reference. The region highlighted by the red and green square boxes is enlarged below to show finer details. Please zoom in for best viewing.

**Figure 5:**
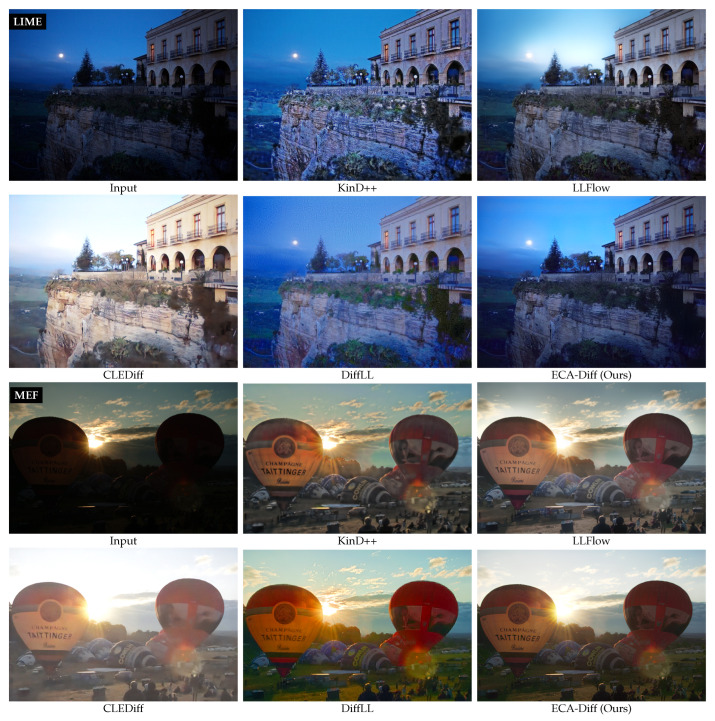

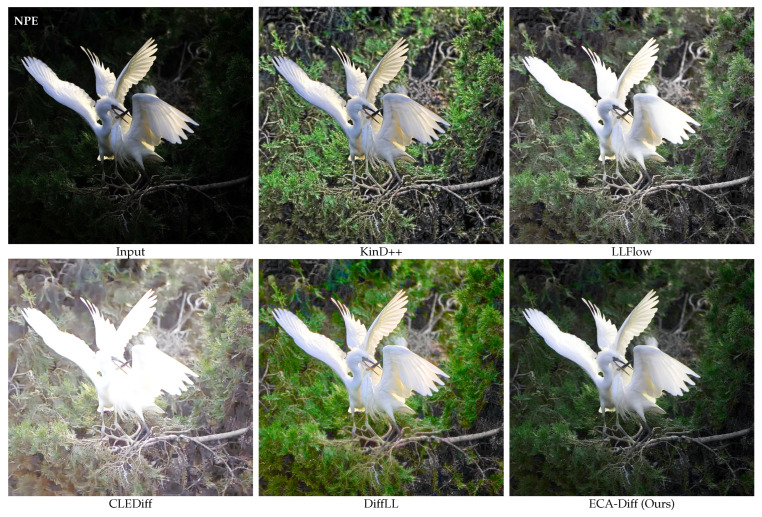
Qualitative comparison on the unpaired benchmarks LIME, MEF, and NPE. For each dataset, one representative image is shown and six methods are displayed in a fixed order: Input, KinD++ [13], LLFlow [18], CLEDiff [21], DiffLL [20], and ECA-Diff (Ours). Dataset labels are overlaid at the top-left of the first panel in each group. Zoom in for best viewing.

Building on the datasets and evaluation protocols detailed in Section 3.6, we present quantitative and qualitative results for ECA-Diff. For the paired benchmarks LOL and LOL-v2 Real, we compare *PSNR*, *SSIM* (higher is better), and *LPIPS* (lower is better), together with visual examples illustrated in Table 1, Figure 3 and Figure 4. For the real-world unpaired sets DICM, LIME, MEF, NPE, and VV, we report no-reference *BRISQUE* and *NIQE* (both lower is better) and show qualitative comparisons in Table 2 and Figure 5. For a detailed comparison of model size and inference latency against representative transformer- and diffusion-based LLIE methods, please refer to Section 5.3 and Table 3.

### 4.1. Paired Benchmarks (LOL/LOL-v2)

**Quantitative Results.** We compare ECA-Diff against state-of-the-art LLIE methods across multiple families: *CNN-based* methods including Zero-DCE [59], RetinexNet [11], and KinD++ [13]; *Transformer-based* methods including SNR-Aware [14], LLFormer [15], RetinexFormer [16], and KAN-T [17]; *Normalizing flow* method LLFlow [18]; *Diffusion-based* methods including DiffLL [20], AnlightenDiff [22], and CLEDiff [21].

Across both paired datasets (LOL and LOL-v2 Real), ECA-Diff achieves state-of-the-art performance on all three metrics (PSNR/SSIM ↑, LPIPS ↓). On LOL, it reaches **27.79** dB PSNR, **0.887** SSIM, and **0.074** LPIPS, exceeding the second-best methods by +0.61 dB PSNR over RetinexFormer (27.18 dB), +0.014 SSIM over LLFlow (0.873), and −0.041 LPIPS over LLFlow (0.115). These gains correspond to higher pixel-level fidelity (PSNR), stronger structural consistency (SSIM), and better perceptual quality (LPIPS). On LOL-v2 real, ECA-Diff attains **28.90** dB PSNR, **0.899** SSIM, and **0.086** LPIPS. The margins are +0.02 dB PSNR over DiffLL (28.88 dB), +0.015 SSIM over KAN-T (0.884), and −0.014 LPIPS over DiffLL (0.100). Although the PSNR margin over DiffLL is small, which suggests comparable fidelity, the consistently higher SSIM and lower LPIPS nevertheless demonstrate stronger detail enhancement and superior perceptual naturalness.

For a fair comparison, we follow the same diffusion framework and training pipeline as CLEDiff when constructing our baseline. Relative to this baseline, ECA-Diff removes the self-attention blocks inside the iterative backbone and instead introduces the external ECAE with TCF-based multi-scale injection. Under these matched settings, ECA-Diff improves CLEDiff by +2.21 dB PSNR/+0.072 SSIM/−0.077 LPIPS on LOL, and by +2.21 dB/+0.070/−0.075 on LOL-v2 real, highlighting the effectiveness of the proposed external context-aware guidance.

**Qualitative Results.** Representative visual comparisons on LOL and LOL-v2 are provided in Figure 3 and Figure 4, respectively. In Figure 3, the first two sets show that ECA-Diff recovers finer details and more faithful colors than competing methods. In the third set (red zoom box), ECA-Diff yields the sharpest digits on the clock and the most consistent background-wall color, closely matching the reference. Upon close inspection of the zoomed-in crops, markedly stronger global noise suppression becomes evident. In Figure 4, ECA-Diff yields, in set 1, cleaner character edges and improved background color; in set 2, it yields clearer restoration of the cloud-like texture, higher wall color fidelity, and more uniform large-scale color across the floor and wall. These gains stem from ECAE’s global context modeling, which stabilizes color, suppresses noise, and preserves fine structures.

### 4.2. Unpaired Benchmarks (DICM/LIME/MEF/NPE/VV)

**Quantitative Results.** On real-world unpaired datasets, we compare ECA-Diff against LIME [4], Zero-DCE [59], EnlightenGAN [60], KinD++ [13], SNR-Aware [14], LLFlow [18], CLEDiff [21], and DiffLL [20]. Quantitative results are summarized in Table 2.

Across the five unpaired datasets, ECA-Diff attains the best score on at least one metric for every dataset, including simultaneous best NIQE and BRISQUE on MEF, best BRISQUE on LIME and VV, and best NIQE on DICM, while remaining competitive elsewhere (second-best NIQE on LIME and VV, and second-best BRISQUE on NPE). Aggregating over datasets, it delivers the best average on both NIQE and BRISQUE. Since NIQE quantifies deviation from natural image statistics and BRISQUE penalizes departures from spatial naturalness (e.g., artifacts and over/under-enhancement), jointly lower values indicate improved global illumination correction with fewer perceptual distortions.

It is worth noting that the no-reference datasets are not used for training, and their content and illumination styles differ significantly from both the paired training sets and from each other. Consequently, NIQE and BRISQUE on these datasets mainly reflect the generalization ability of each method under distribution shift, so their per-dataset rankings may naturally deviate from the full-reference PSNR/SSIM/LPIPS rankings obtained on paired benchmarks. This behavior is further explained by the fact that NIQE and BRISQUE are derived from different natural scene statistics and prefer different trade-offs between smoothness, sharpness, and contrast. In our results, occasional cases where another method achieves a slightly better NIQE or BRISQUE but performs worse on full-reference metrics are consistent with the known biases of these measures and dataset-specific statistics, rather than indicating instability of ECA-Diff. Overall, the full-reference improvements on paired datasets together with the favorable no-reference scores on unseen real-world images demonstrate that ECA-Diff generalizes robustly with a balanced trade-off between fidelity and perceptual naturalness.

**Qualitative Results.** Figure 5 presents qualitative comparisons on LIME, MEF, and NPE, covering scenes with severe underexposure and color casts. Overall, ECA-Diff produces sharper textures, more faithful colors, and fewer artifacts, yielding a more natural and visually comfortable appearance across datasets. In contrast, KinD++ often exhibits over-smoothing together with saturation overshoot; LLFlow tends to break down in dark regions (e.g., tree branches in the NPE example) and shows hue discontinuities in the sky (LIME/MEF); CLEDiff frequently over-exposes bright areas. DiffLL delivers generally pleasing results (e.g., the balloons in MEF) and can rival ours in some views, yet issues remain: oversaturation in green vegetation (LIME/NPE), a cyan/green shift in the MEF sky, and non-smooth artifacts that become apparent upon zoom-in. Together with the no-reference scores in Table 2, these observations substantiate the strong low-light enhancement capability of ECA-Diff on real, unpaired images.

## 5. Ablation Studies

We dissect ECA-Diff along five axes: *(i)* where to inject ECAE within the backbone; *(ii)* which ECAE components contribute most; *(iii)* the efficiency and computational complexity analysis of ECA-Diff; *(iv)* the impact of training patch size to probe global-context learning; and *(v)* the effect of removing LACL objective during training. Unless otherwise specified, all ablation experiments are conducted on the LOL dataset with batch size bs=12 and patch size ps=128. The efficiency comparison is based on fully optimized ECA-Diff, while the patch-size study varies ps with all other settings fixed. We report PSNR and SSIM for quantitative evaluation.

### 5.1. ECAE Placement Across Backbone Stages

This subsection investigates where to inject ECAE into the diffusion backbone and quantifies the accuracy–efficiency trade-off. We follow the U-Net [25] taxonomy and use the placement notation *Down (D)* for the encoder/down-sampling stages, *Mid* for the bottleneck/middle stage, and *Up (U)* for the decoder/up-sampling stages. Unless stated otherwise, efficiency numbers are reported as per-step averages for DDIM sampling with *N* = 100 on full-resolution LOL images (400×600).

As illustrated in Table 4, inserting the ECAE-based context guidance at any backbone stage consistently improves quality over the baseline; mid-stage injection offers the best accuracy–efficiency trade-off, adding the up-sampling stage on top of mid yields the largest absolute gain, and enabling all stages delivers the overall best PSNR/SSIM (26.46/0.864). Although the external encoder increases the number of trainable parameters, the amortized per-step latency grows only slightly because the encoder is executed once and reused across diffusion steps, as indicated by the small gap between *ms/step (image)* and *ms/step (sample)*—most of the overhead arises from per-step feature injection rather than the one-off encoder. Consequently, we adopt the *Full* insertion as the default: with 128×128 training patches it improves the baseline by approximately +0.90 dB PSNR and +0.035 SSIM with an amortized per-step time increase of ≈22.9% (from 157.5 ms to 193.5 ms), alongside a moderate rise in per-step FLOPs.

### 5.2. ECAE Component Analysis

This subsection conducts a hierarchical ablation under the mid-stage injection configuration, denoted as *ECA-Diff (Mid)*; at the macro level we toggle the VAE latent encoder E and LCN, at the micro level we dissect HTC block by enabling or disabling the Transformer branch, the CNN branch, and Bi-CA. Beyond these switches, we further isolate specific components: the external prior of pretrained E with controlled alternatives (Table 5), and the HTC fusion strategies (Bi-CA versus addition/concatenation, Table 6).

**(1) Components Ablations.** From Table 7, three observations emerge: (i) LCN is indispensable, directly injecting the VAE latent into the mid stage without LCN substantially degrades quality, showing that structured context extraction is necessary; (ii) within HTC block, the Transformer and CNN branches are complementary, removing either branch reduces performance; and (iii) disabling the Bi-CA further erodes accuracy, indicating that cross-branch fusion is an effective mechanism for leveraging heterogeneous features. Overall, the complete ECAE injection achieves the best quality among the tested variants; compared with the baseline, *ECA-Diff (Mid)* improves performance by +0.42 dB PSNR and +0.015 SSIM.

**(2) Impact of the Pretrained Latent Encoder.** To assess the benefit of the external prior and the potential domain gap, we evaluate variants under the mid-stage injection setting with LCN enabled (Table 5). The trends are clear: freezing E leads to noticeable degradation, and freezing a pretrained encoder harms performance even more than freezing a randomly initialized one, indicating a mismatch between the pretraining domain and LLIE when reused without adaptation. Allowing E to learn from scratch recovers and slightly improves over the baseline, while fine-tuning a pretrained E consistently achieves the best PSNR/SSIM among all settings. These results show that large-scale pretraining provides useful global semantics, but light task-specific adaptation is necessary to mitigate the domain gap, motivating our default choice of a pretrained and fine-tuned E.

**(3) Effect of Fusion Strategies in the HTC Block.** Table 6 compares three fusion strategies in HTC under the same backbone and mid-stage injection setting. Element-wise addition fails to exploit ECAE and even degrades performance, indicating that naive linear fusion cannot properly align the Transformer branch (global semantics) and the CNN branch (local details). Channel concatenation delivers clear quality improvements over the baseline but increases parameters and runtime due to the wider projection layer. The proposed Bi-CA achieves the best PSNR/SSIM with only a moderate overhead relative to concatenation, showing that explicitly modeling bidirectional interactions between global and local features is a more effective and efficient strategy for context refinement in HTC.

### 5.3. Efficiency and Complexity Analysis

To comprehensively assess the efficiency of ECA-Diff and its design choices, we establish a unified evaluation protocol consisting of: (i) step-count analysis, (ii) overall latency comparison, (iii) comparable-capacity studies, and (iv) condition injection overhead analysis. All measurements are conducted on the LOL dataset with an input resolution of 400×600, a batch size of 1, and a shared hardware/software environment (a single RTX 4090 GPU). We report (i) PSNR/SSIM, (ii) the total number of learnable parameters (M), (iii) the per-step sampling latency of the denoiser $T_{\mathrm{step}}$ (s/step), and (iv) the end-to-end inference latency $T_{\mathrm{image}}$ (s/image). Unless otherwise specified, all efficiency experiments are based on the fully optimized *ECA-Diff (Full)* configuration, whose performance is reported in Table 1.

For standard diffusion-based models without additional one-off modules, the inference latency is well approximated by

(16) $$T_{\mathrm{image}}\approx N\;\cdot \;T_{\mathrm{step}},$$

where *N* is the number of DDIM sampling steps and $T_{\mathrm{step}}$ is the average denoising-step latency. For ECA-Diff, which introduces a once-per-image ECAE, the total latency can be decomposed as

(17) $$T_{\mathrm{image}}\approx T_{\mathrm{ECAE}}+N\;\cdot \;T_{\mathrm{step}},$$

where $T_{\mathrm{ECAE}}$ denotes the one-off ECAE cost and $T_{\mathrm{step}}$ is measured from the iterative sampler alone. In practice, Equations (16) and (17) closely matches the observed runtimes, with minor deviations due to data transfer, kernel scheduling, and measurement noise. For single-pass Transformer-based baselines (*N* = 1) without iterative sampling, we directly report the end-to-end latency as $T_{\mathrm{image}}$.

**(1) Effect of DDIM Steps.** Table 8 reports the behavior of ECA-Diff under different DDIM sampling steps *N*. With very few steps (*N* = 5), the model is clearly under-converged, leading to ineffective denoising and significant quality degradation. From *N* = 10 onward the performance improves substantially, and starting at *N* = 25 the results become highly stable: for *N* = 25, 50, 75, 100, both PSNR and SSIM fluctuate only marginally, indicating that ECA-Diff quickly saturates once a moderate number of steps is used. The sampling time $T_{\mathrm{sample}}$ grows approximately linearly with *N*, with a nearly constant per-step sampling cost around 0.18–0.20 s. The one-time ECAE overhead (latent encoder E plus LCN) is about 0.07 s per image, which remains small compared with the cumulative sampling cost in typical multi-step settings and is well captured by Equation (17). In the main quantitative comparisons (Table 1), we adopt a conservative default of *N* = 100 steps to report a stable high-quality configuration, while the above results show that ECA-Diff maintains competitive performance even with reduced step counts (e.g., *N* = 25 or 50).

**(2) Overall Latency Comparison.** Table 3 compares representative transformer-based and diffusion-based LLIE methods on LOL. For transformer-based methods (SNR-Aware [14], RetinexFormer [16]), $T_{\mathrm{image}}$ is the direct single-pass latency. For diffusion-based methods (DiffLL [20], PyDiff [19], CLEDiff [21]), we list the default iterative number *N*, the measured end-to-end latency $T_{\mathrm{image}}$, and the average per-step sampling cost $T_{\mathrm{step}}$. For methods without additional one-off modules, $T_{\mathrm{step}}$ closely matches $T_{\mathrm{image}}/N$; for ECA-Diff, $T_{\mathrm{step}}$ is obtained from the isolated sampler runtime and the total latency follows Equation (17).

The transformer baselines achieve low latency thanks to their lightweight architectures and single-step inference, with RetinexFormer reaching 27.18 dB PSNR using only 1.61 M parameters. Diffusion-based LLIE models offer stronger generative capacity, but their absolute latency is heavily influenced by backbone design and step scheduling. DiffLL exploits wavelet-domain diffusion and PyDiff adopts a pyramid formulation with only four sampling steps, both of which explicitly optimize the sampling process for efficiency. Under this setting, our ECA-Diff, built upon a classical DDPM-style framework with standard DDIM sampling, is not directly tailored to the same extreme-latency regime as these specialized designs.

Our focus here is on the efficiency of the proposed ECA-Diff framework itself. CLEDiff serves as a strong and fair baseline, since it follows the same diffusion paradigm and training strategy. Compared with CLEDiff, ECA-Diff removes the self-attention blocks in the iterative backbone and offloads context modeling to an external, once-executed ECAE, which is coupled back through lightweight TCF modules. Although this redesign roughly doubles the total parameter count (from 84.88 M to 105.74 M + 61.68 M), it yields both faster sampling and higher restoration quality. With *N* = 10 steps, ECA-Diff reduces the per-step latency from 0.224 s to 0.179 s (about 20% reduction) and the end-to-end latency from 2.240 s to 1.857 s (about 17% reduction), while improving performance by +2.13 dB PSNR and +0.056 SSIM. With *N* = 100 steps, the per-step cost still decreases (0.224 s → 0.192 s, ∼14% reduction), and ECA-Diff achieves gains of +2.21 dB PSNR and +0.072 SSIM over CLEDiff. These results indicate that reallocating capacity into an external encoder and simplifying the recurrent denoising pathway leads to a more efficient diffusion framework, even when all parameters are accounted for. To further disentangle the benefits of the proposed framework from pure model scaling, the next subsection evaluates ECA-Diff under comparable-capacity settings against CLEDiff and related baselines.

It is also worth noting that the proposed ECA-Diff is orthogonal to backbone-lightening and step-reduction techniques used by DiffLL and PyDiff. Since ECAE and TCF are designed as plug-in components on top of a standard DDPM backbone, they can in principle be combined with wavelet-domain parameterization, pyramid formulations, or other advanced sampling schedules, opening up additional room for jointly improving both efficiency and restoration quality.

**(3) Efficiency Under Comparable Capacity.** In this study, we compare ECAE against backbones that *increase capacity without using ECAE* so as to attain *comparable capacity* (i.e., similar parameter count and/or per-step FLOPs). We use ch_mult to denote the stage-wise channel multipliers of the U-Net encoder–decoder, and attn_use to list the stages (1-indexed) where global attention (GA) blocks are inserted. For a fair comparison, all models listed in Table 9, including *ECA-Diff (Full)*, are trained from scratch under the same setting (bs=12, ps=128 for 8000 rounds). We report PSNR/SSIM together with model size, FLOPs per step, and $T_{\mathrm{image}}$ under DDIM sampling with *N* = 100 on full-resolution LOL images (400×600).

From Table 9 it is clear that enlarging the backbone alone does not guarantee higher LLIE quality. The *Deeper* model approximately doubles the parameter count (83.1 M → 179.1 M; +115%) and slightly increases per-step FLOPs from 3400 G to 3593 G (+5.7%), yet its accuracy decreases to 24.93/0.823, indicating that the improvement delivered by our method arises from task-aligned design rather than model capacity. Increasing capacity further with *Deeper + Wider + GA* strengthens long-range modeling, but the quality remains below *ECA-Diff (Full)* (25.31/0.833 compared with 26.46/0.864) despite similar compute (4286 G and 4417 G FLOPs per step). In addition, adding GA to shallow, large-resolution stages leads to a much higher per-image latency (27.59 s), whereas *ECA-Diff (Full)* maintains a lower per-image latency (19.35 s) by computing global context once in the latent space and reusing it across diffusion steps. Overall, we conclude that the superior performance-efficiency of ECA-Diff results from external, one-off context extraction with lightweight per-step injection rather than brute-force increases in parameters or FLOPs.

**(4) TCF vs. LDM-style Conditioning.** Table 10 compares three context injection strategies under incremental multi-stage injection. To focus on the fusion overhead, we report backbone parameters and the per-step smpling latency $T_{\mathrm{step}}$, excluding the one-time ECAE cost. In the LDM-style token cross-attention (CA) scheme, the ECAE output is compressed into a fixed 16×16 token map and each selected stage performs cross-attention with these tokens. Adding more injection stages accumulates additional cross-attention blocks over multi-scale features, leading to a steady increase in $T_{\mathrm{step}}$ from the mid-only setting to the full M + U configuration. In the spatial CA scheme, ECAE features are resized to match each stage and full-resolution cross-attention is applied in the spatial domain. The complexity grows rapidly with spatial size, causing a sharp rise in $T_{\mathrm{step}}$ when multiple decoder stages are enabled and resulting in out-of-memory for deeper multi-stage variants.

The proposed TCF instead aligns ECAE features with backbone resolutions and fuses them via lightweight time modulation and channel concatenation, without explicit attention. Its cost scales approximately linearly with the number of injection stages, and the *ECA-Diff (M + U)* configuration maintains a per-step latency of 0.180 s with 99.18 M backbone parameters, substantially lower than the LDM-style and spatial CA counterparts. This contrast is consistent with the original motivation of LDM-style cross-attention, which is efficient when the backbone operates in a low-resolution latent space with fixed-length text tokens, but becomes less suitable for full-resolution, detail-sensitive LLIE; TCF better matches the design characteristics of ECA-Diff by providing scalable external guidance with well-controlled overhead.

### 5.4. Impact of Patch Size

We study how the training patch size ps influences performance for both the baseline and ECA-Diff. Since ECAE aggregates global, context-aware information, our hypothesis is that larger patches expose longer-range statistics and thus benefit ECA-Diff more than a baseline without explicit global modeling. To test this, we compare the two models at ps∈{64,128,256}, as summarized in Table 11. To isolate the effect of ps, all other training and architecture settings are kept fixed. The batch size bs is scaled approximately as $1/ps^{2}$ so that the per-step pixel throughput $bs\times ps^{2}$ is nearly constant. The number of epochs is adjusted to keep the total optimizer updates unchanged.

Table 11 shows that enlarging the training patch improves baseline PSNR, while SSIM remains unstable and even drops at ps = 256, indicating that without explicit global context modeling larger crops mainly enhance pixel fidelity but do not consistently preserve structure. For ECA-Diff, the gain at small patches is negligible at ps = 64 because ECAE downsamples five times and the latent grid is only 2×2, which limits the amount of recoverable global context; as ps increases to 128 and 256, the latent space carries richer scene statistics and quality rises rapidly, surpassing the baseline by +0.90 dB PSNR and +0.035 SSIM at ps = 128, and by +1.29 dB PSNR and +0.079 SSIM at ps = 256. To obtain the best accuracy while keeping training efficient, we adopt the two-stage schedule 128 → 256, which produces the final results reported in this paper.

### 5.5. Ablation on the LACL

**Figure 6:**
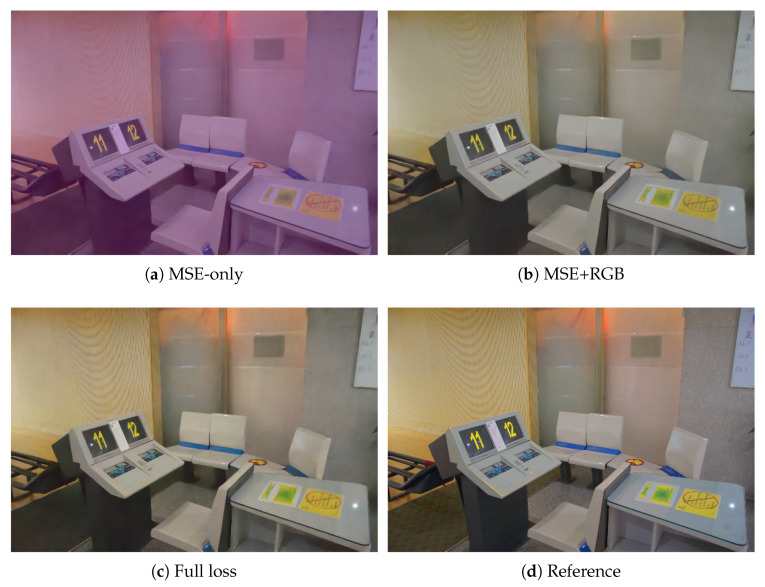
Qualitative comparison aligned with Table 12. Please focus on the restoration of tone and chroma over large regions.

To verify the effectiveness of the LACL term with minimal confounders, we start from the mid-stage configuration *ECA-Diff (Mid)* and vary only the training objective in Equation (15). We compare three settings: *MSE-only*, which uses only the noise-prediction objective $L_{\mathrm{ddpm}}^{\mathrm{cond}}$; *MSE + RGB*, which additionally enables the RGB-space terms $L_{\mathrm{RGB}-\ell_{1}}$, $L_{\mathrm{SSIM}}$, and $L_{\mathrm{perc}}$; and *Full loss*, which further includes the LACL term $L_{\mathrm{LACL}}$. As reported in Table 12, RGB-space supervision improves upon MSE-only, and adding $L_{\mathrm{LACL}}$ yields the best and most consistent PSNR/SSIM, indicating that LACL provides complementary guidance beyond pixel and perceptual matching. Qualitatively, as shown in Figure 6, *MSE-only* produces severe color bias with noticeable hue shifts and distorted chroma in neutral regions. Enabling the RGB-space losses (*MSE + RGB*) markedly improves structure and exposure, yet introduces a clear cool color cast that persists across large areas. Incorporating LACL (*Full loss*) effectively suppresses this cool tint, restoring neutral whites and more faithful hues; the resulting tone and chroma are closest to the reference image.

## 6. Discussion

**Overall Performance.** The experimental results demonstrate that the proposed ECA-Diff framework effectively strengthens diffusion-based LLIE by introducing an external, compute-once global context pathway. Compared with recent CNN-, Transformer-, and diffusion-based approaches, ECA-Diff provides consistent gains on paired benchmarks; under similar diffusion backbone and training pipeline as CLEDiff, it yields notable improvements in PSNR, SSIM, and LPIPS on LOL and LOL-v2 Real, and the efficiency and complexity analysis under parameter- and FLOPs-matched settings further indicates that these gains are attributable to external context guidance rather than increased backbone capacity. On real-world unpaired datasets, ECA-Diff achieves the best or highly competitive BRISQUE and NIQE scores while producing visually stable illumination and color, suggesting that the injected context helps align the generative behavior of diffusion models with natural image statistics instead of over-amplifying contrast or saturation.

**Model Design.** From a design perspective, ECAE decouples global context estimation from the iterative denoising loop: deep context features are computed once in a compact latent space and reused across all steps, which reduces the potential marginal per-step overhead and alleviates conflicts between local denoising and global correction. Ablation results confirm that effective multi-stage injection is essential; naive reuse of a frozen pretrained latent encoder or simple additive fusion is suboptimal, whereas a pretrained-and-fine-tuned latent encoder, an LCN built with hybrid CNN/Transformer branches, and Bi-CA collectively provide stable and complementary gains. Within this framework, LCN captures long-range dependencies in the latent space, and TCF injects the resulting multi-scale context into the backbone through lightweight, timestep-aware modulation, avoiding the quadratic cost of full-resolution cross-attention. The unified efficiency and complexity analysis shows that these benefits are achieved with moderate overhead, indicating that the performance gains stem from targeted, cost-effective architectural choices rather than brute-force model scaling. The LACL objective complements this design by regularizing chromaticity in CIELAB space, mitigating the typical cool, desaturated bias in dark regions and promoting plausible, stable color reproduction.

**Figure 7:**
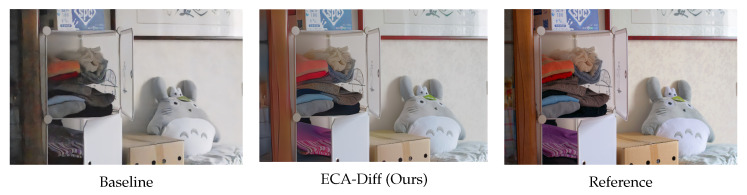
Visual comparison on a challenging indoor scene. ECA-Diff suppresses noise and corrects global illumination compared with the baseline, but shows slightly over-smoothed textures and mildly over-enhanced saturation relative to the reference (please zoom in for a clearer view), illustrating the trade-off between strong denoising with stable color and faithful preservation of fine details.

**Limitations and failure cases.** Despite these advantages, several limitations remain. First, the overall inference time is still constrained by the iterative nature of the DDPM-style backbone: ECA-Diff keeps the additional cost modest by computing the external context once and injecting it via lightweight TCF modules, but does not match the absolute runtime of one-pass lightweight LLIE networks or diffusion models with aggressively accelerated samplers or distilled backbones. Second, ECA-Diff can be challenged in extremely low SNR scenes, under strong spatially varying color casts, or with dense fine-grained textures. As illustrated in Figure 7, it effectively suppresses noise and corrects global illumination relative to the baseline diffusion model, but may yield slightly over-smoothed textures and mildly over-enhanced saturation, reflecting a bias toward clean, color-consistent solutions when local evidence is weak and revealing an inherent trade-off between robustness and perfect preservation of micro-structures.

**Future Directions.** From a broader perspective, this work highlights the potential of combining task-specific external encoders with diffusion models for low-level vision. Although ECA-Diff is instantiated on a vanilla DDPM-style backbone and thus inherits relatively high absolute runtime, our experiments validate the effectiveness of the compute-once external context paradigm and show that it is orthogonal to backbone and sampling accelerations. A primary direction is therefore to couple ECA-Diff with accelerated diffusion samplers, distilled or lightweight backbones, and other efficiency-oriented designs to better match the training and inference efficiency of emerging diffusion architectures while further improving the quality–efficiency trade-off. In parallel, extending the concept of ECAE and the compute-once, multi-step conditioning scheme to other restoration tasks such as deblurring, dehazing, inpainting, and super-resolution is a natural step, as these problems also benefit from robust global structural cues. Another promising avenue is to design adaptive context encoders that explicitly model sensor characteristics and scene semantics for more reliable enhancement in extremely dark or mixed-illumination environments.

## 7. Conclusions

We presented ECA-Diff, which enhances diffusion-based LLIE by externalizing global-context estimation via a task-specific encoder and reusing it as step-shared conditioning for the denoising backbone. This decoupled integration yields consistent improvements in fidelity and perceptual quality while adding only a controllable, sampling-dependent latency. In addition, we introduce LACL in CIELAB color space that emphasizes darker and more colorful regions while de-emphasizing near-gray pixels, thereby regularizing diffusion training toward accurate hue and natural saturation. The proposed compute-once, multi-step conditioning paradigm is broadly applicable to low-level restoration tasks. Future work will focus on accelerating the ECAE and its injection pipeline and on extending the paradigm to deblurring, dehazing, and super-resolution.

## Figures and Tables

**Table 1:** Paired quantitative results on LOL and LOL-v2 Real. Metrics are PSNR/SSIM ↑ (higher is better) and LPIPS ↓ (lower is better). Best is **red bold**; second best is blue underlined. A dash (“–”) denotes unavailable results. Scores are taken from original papers or reproduced using authors’ official pretrained models.

Method	Model Type	LOL			LOL-v2 Real		
		PSNR ↑	SSIM ↑	LPIPS ↓	PSNR ↑	SSIM ↑	LPIPS ↓
Zero-DCE [59]	CNN	14.86	0.562	0.335	18.06	0.580	0.313
RetinexNet [11]	CNN	16.77	0.462	0.474	18.371	0.723	0.365
KinD++ [13]	CNN	21.30	0.823	0.175	19.09	0.817	0.180
SNR-Aware [14]	Transformer	26.72	0.851	0.152	27.21	0.871	0.158
LLFlow [18]	Normalizing flow	25.18	0.873	0.115	25.54	0.876	0.156
LLFormer [15]	Transformer	25.76	0.823	0.167	26.20	0.819	0.209
RetinexFormer [16]	Transformer	27.18	0.850	0.129	27.70	0.856	0.166
AnlightenDiff [22]	Diffusion	21.73	0.814	0.141	20.66	0.837	0.146
CLEDiff [21]	Diffusion	25.58	0.815	0.151	26.69	0.829	0.161
DiffLL [20]	Diffusion	26.91	0.847	0.119	28.88	0.874	0.100
KAN-T [17]	Transformer	26.66	0.854	–	28.45	0.884	–
**ECA-Diff (Ours)**	Diffusion	**27.79**	**0.887**	**0.074**	**28.90**	**0.899**	**0.086**

**Table 2:** No-reference quantitative results on real-world unpaired benchmarks. Metrics are NIQE ↓ and BRIS ↓ (*BRIS* abbreviates *BRISQUE*); ↓ indicates that lower is better. AVG. denotes the average across all datasets. Best and second best results are indicated by **red bold** and blue underlined text, respectively.

Method	DICM		LIME		MEF		NPE		VV		AVG.	
	NIQE ↓	BRIS ↓	NIQE ↓	BRIS ↓	NIQE ↓	BRIS ↓	NIQE ↓	BRIS ↓	NIQE ↓	BRIS ↓	NIQE ↓	BRIS ↓
LIME [4]	4.48	27.38	5.05	32.84	4.74	39.10	4.17	28.94	3.71	18.93	4.43	29.44
Zero-DCE [59]	3.95	23.35	4.38	26.05	3.50	29.36	3.83	21.84	5.08	21.84	4.15	24.49
EnlightenGAN [60]	3.83	19.13	4.25	22.66	3.56	26.80	3.78	21.16	3.69	14.15	3.82	20.78
KinD++ [13]	3.86	26.29	6.38	28.89	3.78	27.09	4.41	18.91	**2.68**	21.90	4.22	24.62
SNR-Aware [14]	3.80	19.46	4.60	29.02	4.06	28.33	3.94	28.42	3.76	23.67	4.03	25.78
LLFlow [18]	3.83	24.72	7.36	30.71	3.97	29.75	4.08	20.01	3.18	30.09	4.48	27.06
CLEDiff [21]	4.51	25.43	4.57	22.27	4.70	26.51	4.84	22.75	4.33	22.57	4.59	23.91
DiffLL [20]	3.81	**18.58**	**3.78**	19.84	3.43	24.17	**3.43**	**15.79**	3.51	14.64	3.59	18.6
**ECA-Diff (Ours)**	**3.75**	23.84	3.99	**17.99**	**3.24**	**16.39**	3.97	18.57	2.94	**10.53**	**3.58**	**17.46**

**Table 3:** Comparison of LLIE performance (PSNR/SSIM ↑), model complexity, and inference efficiency on LOL. $T_{\mathrm{image}}$ denotes the average end-to-end inference time per image. For diffusion-based methods with *N* steps, we also report the per-step sampling latency $T_{\mathrm{step}}$. **Bold** values denote the best results.

Method	Type	PSNR ↑	SSIM ↑	Params (M)	*N*	$T_{\mathrm{image}}$ (s)	$T_{\mathrm{step}}$ (s)
SNR-Aware	Transformer	26.72	0.851	39.12	1	0.036	0.036
RetinexFormer	Transformer	27.18	0.850	1.61	1	0.076	0.076
DiffLL	Diffusion	26.91	0.847	22.08	10	0.490	0.049
PyDiff	Diffusion	27.18	0.882	97.89	4	1.514	0.379
CLEDiff (10 steps)	Diffusion	25.27	0.765	84.88	10	2.240	0.224
CLEDiff (100 steps)	Diffusion	25.58	0.815	84.88	100	22.392	0.224
ECA-Diff (10 steps)	Diffusion	27.40	0.821	105.74 + 61.68 ^†^	10	1.857	0.179
ECA-Diff (100 steps)	Diffusion	**27.79**	**0.887**	105.74 + 61.68 ^†^	100	19.351	0.192

^†^ For ECA-Diff, 105.74 M parameters belong to the diffusion backbone with TCF modules, while 61.68 M parameters correspond to the once-executed ECAE, including the latent encoder E and the LCN.

**Table 4:** ECAE insertion study on LOL. Quality is reported with PSNR/SSIM ↑. Efficiency columns are *per-step averages* for DDIM sampling at *N* = 100. *FLOPs/step* and *ms/step (image)* include the one-off ECAE amortized over steps, whereas *ms/step (sample)* excludes the one-off encoder. ✓ indicates insertion of ECAE at the corresponding stage; **bold** values denote the best results.

Variant	ECAE Insertion Stages			Quality		Model Size	Efficiency (per DDIM Step @ *N* = 100)		
	Enc (Down)	Mid	Dec (Up)	PSNR ↑	SSIM ↑	Params (M)	FLOPs/ Step (G)	ms/Step (Sample)	ms/Step (Image)
Baseline				25.56	0.829	83.11	3400	157.5	157.5
ECA–Diff (Mid)		✓		25.98	0.844	148.5	3420	157.6	158.6
ECA–Diff (D + M)	✓	✓		26.12	0.830	156.4	3826	172.1	173.2
ECA–Diff (M + U)		✓	✓	26.25	0.858	160.9	4029	179.6	180.4
**ECA–Diff (Full)**	✓	✓	✓	**26.46**	**0.864**	167.4	4417	192.4	193.5

**Table 5:** Impact of the latent encoder E pretraining under *ECA-Diff (Mid)* on LOL. All rows except the baseline use ECAE with LCN trained from scratch; only E training states differ. Init: Rand = random init; Pre = pretrained. Train: Frz = frozen; Scratch = train from scratch; FT = fine-tune. Metrics: PSNR/SSIM ↑. **Bold** values denote the best results.

Variant	ECAE			Quality	
	E Init	E Train	LCN Train	PSNR ↑	SSIM ↑
Baseline	N/A	N/A	N/A	25.56	0.829
$V_{1}$	Rand	Frz	Scratch	25.49	0.830
$V_{2}$	Rand	Scratch	Scratch	25.59	0.829
$V_{3}$	Pre	Frz	Scratch	25.42	0.804
$V_{4}$ (Ours)	Pre	FT	Scratch	**25.98**	**0.844**

**Table 6:** Comparison of fusion strategies in the HTC block under *ECA-Diff (Mid)* on LOL. Reported *Params* and *Runtime* refer *only* to the ECAE module (external context); runtime is per-image. “Add” denotes element-wise addition; “Concat” denotes channel concatenation. Metrics: PSNR/SSIM ↑. **Bold** values denote the best results.

Variant	PSNR ↑	SSIM ↑	Params (M)	Runtime (ms)
Baseline (w/o ECAE)	25.56	0.829	N/A	N/A
HTC w/Add	25.34	0.812	26.24	54.67
HTC w/Concat	25.76	0.837	26.49	60.71
HTC w/*Bi-CA* (ours)	**25.98**	**0.844**	27.52	67.94

**Table 7:** Component ablation of ECAE under *ECA-Diff (Mid)* on LOL. Columns are organized hierarchically: *Macro components* (VAE latent encoder E; LCN) and *HTC components*. A ✓ indicates that the component is enabled. When only a single branch is present (w/o Trans or w/o CNN), Bi-CA is not applicable. Metrics: PSNR/SSIM ↑. **Bold** values denote the best results.

Variant	Macro Components		HTC Components			Quality	
	E	LCN	Trans	CNN	Bi-CA	PSNR↑	SSIM↑
Baseline						25.56	0.829
w/o LCN (E → Mid)	✓					25.48	0.818
w/o Transformer branch	✓	✓		✓		25.60	0.835
w/o CNN branch	✓	✓	✓			25.50	0.838
w/o Bi-CA	✓	✓	✓	✓		25.76	0.837
**ECA-Diff (Mid)**	✓	✓	✓	✓	✓	**25.98**	**0.844**

**Table 8:** Effect of DDIM sampling steps on ECA-Diff on LOL. Metrics are PSNR/SSIM ↑. $T_{\mathrm{sample}}=T_{\mathrm{step}}\times N$ denotes the diffusion sampling time per image excluding the one-time ECAE cost. **Bold** values denote the best results.

Steps *N*	PSNR ↑	SSIM ↑	$T_{\mathrm{image}}$ (s)	$T_{\mathrm{sample}}$ (s)	$T_{\mathrm{step}}$ (s)
5	19.25	0.219	0.905	0.834	0.167
10	27.40	0.821	1.857	1.790	0.179
25	**27.93**	0.876	4.746	4.684	0.187
50	27.87	**0.887**	9.598	9.531	0.191
75	27.82	0.887	14.822	14.761	0.197
100	27.79	0.887	19.351	19.244	0.192

**Table 9:** Efficiency under comparable capacity. Quality is PSNR/SSIM ↑. Efficiency numbers (Params and FLOPs) are *per-step averages* for DDIM sampling at *N* = 100. “ECAE injection” uses ✓ for enabled and ✗ for disabled. [ ] denotes none GA blocks are used. **Bold** values denote the best results.

Variant	Backbone Setup		ECAE Injection	Quality		Efficiency		
	ch_mult	attn Use		PSNR↑	*SSIM*↑	Params (M)	FLOPs/Step (G)	$T_{\mathrm{image}}$ (s)
Baseline	[1, 2, 3, 4]	[ ]	✗	25.56	0.829	83.11	3400	15.75
Deeper	[1, 2, 3, 4, 6]	[ ]	✗	24.93	0.823	179.11	3593	16.67
Deeper + Wider + GA	[1, 2, 4, 4, 5]	[2, 3, 4]	✗	25.31	0.833	182.93	4286	27.59
**ECA-Diff (Full)**	[1, 2, 3, 4]	[ ]	✓	**26.46**	**0.864**	167.40	4417	19.35

**Table 10:** Comparison of context injection strategies under incremental multi-stage injection on LOL (400×600). “M” denotes mid-level injection; “U [*k*]” denotes injection at the *k*-th upsampling stage. $T_{\mathrm{step}}$ is the per-step sampling latency (s), excluding the one-time ECAE cost. “OOM” indicates out-of-memory. **Bold** values denote the best results.

Injection Stages	LDM-Style Token CA		Spatial CA		Proposed TCF	
	Params (M)	$T_{\mathrm{step}}$ (s) ↓	Params (M)	$T_{\mathrm{step}}$ (s) ↓	Params (M)	$T_{\mathrm{step}}$ (s) ↓
M	82.49	**0.158**	83.12	0.164	85.77	**0.158**
M + U [0]	84.72	**0.160**	86.29	0.176	92.90	**0.160**
M + U [0, 1]	86.70	0.166	88.08	0.338	96.93	**0.164**
M + U [0, 1, 2]	88.44	0.192	88.88	OOM	98.72	**0.172**
M + U [0, 1, 2, 3]	89.92	0.278	89.08	OOM	99.18	**0.180**

**Table 11:** Impact of training patch size ps on quality. Metrics are PSNR/SSIM ↑. Here ps denotes the training crop size of ps×ps. The last column (128→256) indicates a two-stage schedule: first train with ps = 128 until convergence, then fine-tune with ps = 256; this setting is used for our final reported results. **Bold** values denote the best results.

Metric	Baseline			ECA-Diff (Full)			
	64	128	256	64	128	256	128→256
PSNR ↑	24.84	25.56	26.14	24.96	26.46	27.43	**27.79**
SSIM ↑	0.804	0.829	0.795	0.792	0.864	0.874	**0.887**

**Table 12:** LACL loss ablation on *ECA-Diff (Mid)*. Metrics are PSNR/SSIM ↑. “MSE-only”: $L_{\mathrm{ddpm}}^{\mathrm{cond}}$; “MSE + RGB”: $L_{\mathrm{ddpm}}^{\mathrm{cond}}$ + RGB-space losses $\left\{L_{\mathrm{RGB}-\ell_{1}},L_{\mathrm{SSIM}},L_{\mathrm{perc}}\right\}$; “Full loss”: $L_{\mathrm{train}}$ in Equation (15). **Bold** values denote the best results.

Metric	MSE-Only	MSE + RGB	Full Loss
PSNR ↑	18.84	25.44	**25.98**
SSIM ↑	0.783	0.758	**0.844**

## Data Availability

The publicly archived datasets can be found at https://github.com/weichen582/RetinexNet (LOL dataset, accessed on 15 November 2024), https://github.com/flyywh/CVPR-2020-Semi-Low-Light (LOL-v2 dataset, accessed on 15 November 2024), and https://github.com/baidut/BIMEF (unpaired datasets VV, LIME, NPE, DICM and MEF, accessed on 15 November 2024).

## Abbreviations

The following abbreviations are used in this manuscript:

Bi-CA	Bidirectional Cross-Attention
CA	Cross-Attention
DDIM	Denoising Diffusion Implicit Model
DDPM	Denoising Diffusion Probabilistic Models
ECA-Diff	External Context-Aware encoder guided Diffusion framework
ECAE	External Context-Aware Encoder
FiLM	Feature-wise Linear Modulation
GA	Global Attention
HE	Histogram Equalization
HTC	Hybrid Transformer-Convolution block
IQA	Image Quality Assessment
LACL	Luminance-Adaptive Chromaticity Loss
LCN	Latent Context Network
LDM	Latent Diffusion Models
LLIE	Low-Light Image Enhancement
LPIPS	Learned Perceptual Image Patch Similarity
MSE	Mean Squared Error
PSNR	Peak Signal-to-Noise Ratio
SNR	Signal-to-Noise Ratio
SSIM	Structural Similarity Index Measure
TCF	Time-modulated Context Fusion
VAE	Variational Auto Encoder
